# Supplementary material for: RIPpore: A Novel Host-Derived Method for the Identification of Ricin Intoxication through Oxford Nanopore Direct RNA Sequencing
Source: Toxins (Basel). 2022 Jul 9;14(7):470. doi: 10.3390/toxins14070470 (PMC9319349; doi:10.3390/toxins14070470)
Supplement: Supplementary file 1 [file toxins-14-00470-s001.zip › toxins-1799166-supplementary.pdf]

Supplementary Materials: RIPpore: a Novel Host-Derived Method for the Identification of Ricin Intoxication through Oxford Nanopore Direct RNA Sequencing

Yan Ryan, Abbie Harrison, Hannah Trivett, Catherine Hartley, Jonathan David, Graeme C. Clark and Julian A. Hiscox

Table S1. Overview of Flow cell and direct RNA reads.

Flow cell	Total Reads	Samples Loaded
Control 1	441184	1
Control 2	79569	1
Control 3	14500	1
B-chain	296000	4
Ricin Cell 1	91437	3
Ricin Cell 2	556362	4
Ricin Cell 3	651134	4
Poreplex Cell	192358	4
Saporin Cell	308952	4

Scheme S1. Flow cells show a range of 15,000 to 600,000 reads, with no discernible effect from barcoding on final number of reads.

Table S2. Overview of Sample Reads per sample and reads at RIP depurination site.

Sample	Total Reads Per Sample	Reads at Adenine 4605	Percentage Reads 28S	Barcoding
Control 1	441184	324033	73.4	Single Sample
Control 2	79569	51190	64.3	Single Sample
Control 3	14500	10901	75.2	Singe Sample
Five Hour Ricin	449858	298160	66.3	Single Sample
B-chain 1	26477	16598	62.7	DeePlexiCon
B-chain 2	89836	66066	73.5	DeePlexiCon
B-chain 3	60390	33288	55.1	DeePlexiCon
B-chain 4	96088	72406	75.4	DeePlexiCon
Ricin Cell 1 2 Hours	21977	9913	45.1	DeePlexiCon
Ricin Cell 1 4 Hours	45016	27943	62.1	DeePlexiCon
Ricin Cell 1 6 Hours	13501	6286	46.6	DeePlexiCon
Ricin 24 Hours				
Poreplex	17108	4939	28.9	Poreplex
Saporin 1	39688	5433	13.7	DeePlexiCon
Saporin 2	114318	22022	19.3	DeePlexiCon
Saporin 3	30410	4669	15.4	DeePlexiCon
Saporin 4	106322	27482	25.8	DeePlexiCon
Ricin Cell 2 2 Hours	116094	27839	24.0	DeePlexiCon
Ricin Cell 2 4 Hours	248554	77606	31.2	DeePlexiCon
Ricin Cell 2 6 Hours	94460	21372	22.6	DeePlexiCon
Ricin Cell 2 24 Hours	64504	14745	22.9	DeePlexiCon
Ricin Cell 3 2 Hours	103890	21716	20.9	DeePlexiCon
Ricin Cell 3 4 Hours	269886	79390	29.4	DeePlexiCon
Ricin Cell 3 6 Hours	115988	26668	23.0	DeePlexiCon
Ricin Cell 3 24 Hours	123780	26035	21.0	DeePlexiCon

Table S2. Samples show generally the same order of magnitude of reads with two saporin samples, and one ricin sample in flow cells 2 and 3 being an order of magnitude lower than the remaining reads. The efficiency of the custom barcode ligation to 28S ribosomal reads has a high variance between 15 and 75% but an average of 41% as seen by percentage Reads 28S. .

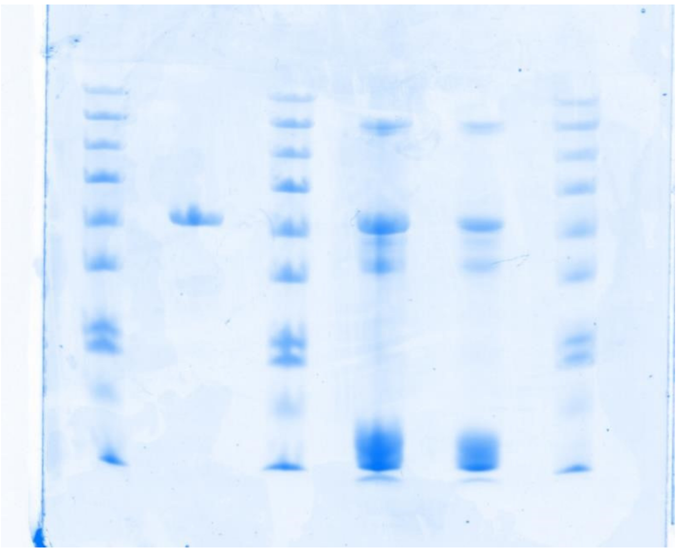

Figure S1. Unedited image of Fig. 2(A) in original orientation. Shows Coomassie blue analysis of crude (right) and purified (left) ricin toxin preparations alongside protein ladders (10-180 kDa).

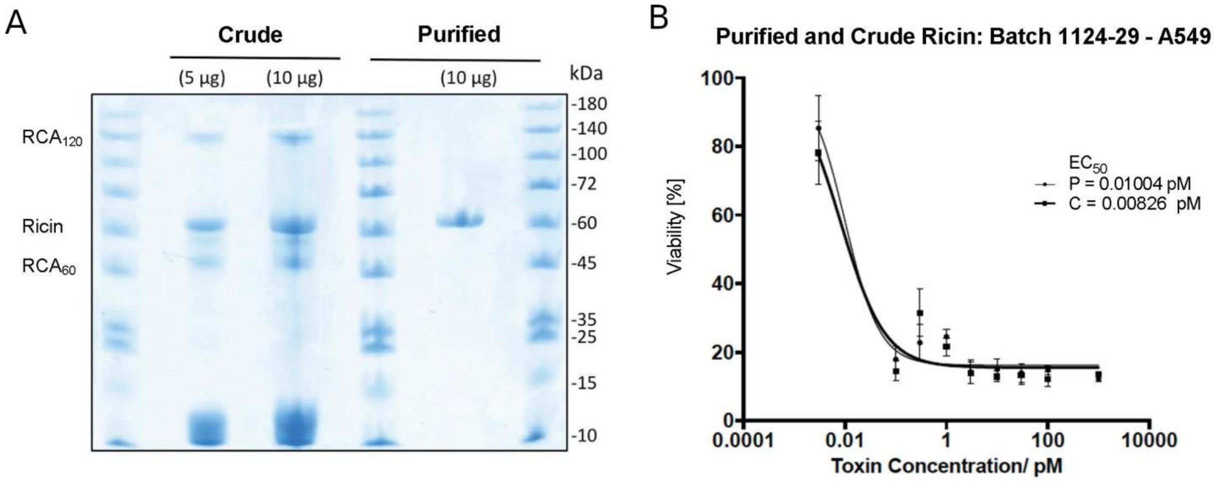

Figure S2. (A). Coomassie blue analysis of crude and purified ricin toxin preparations. Two different amounts of the crude preparation were evaluated (5 g and 10 g) and a single preparation of the purified fraction (10 g). Molecular weight markers are indicated to the right and the expected size of purified ricin and the R. communis agglutinins (RCA120 and RCA60) are indicated to the left. (B). WST-1 viability assay on A549 cells exposed to increasing concentrations of crude (C) and purified (P) ricin toxin for 48 hours showed a plateau in toxicity beginning at 10 pM and a difference in EC50 of 2 fM.

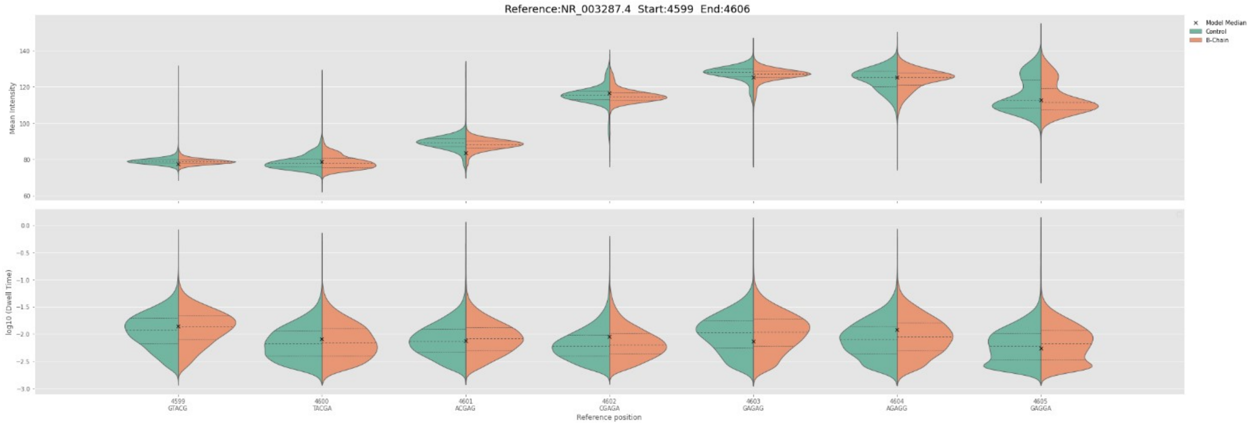
Reference:NR_003287.4 Start:4599 End:4606
Model Median
Control
B-Chain
Mean Intensity
log10 (Dwell Time)
4599 GTACG
4600 TACGA
4601 ACGAG
4602 CGAGA
4603 GAGAG
4604 AGAGG
4605 GAGGA
Reference position

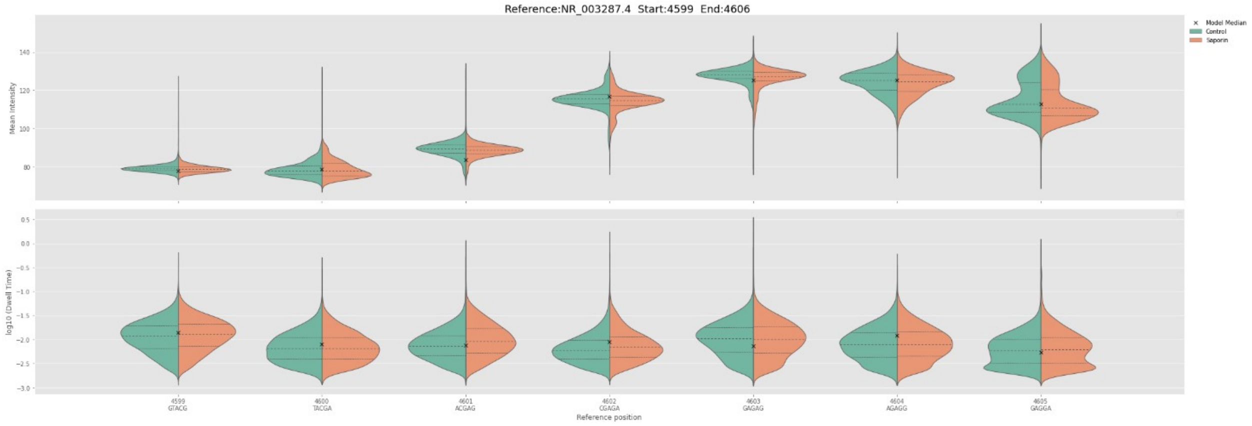
Reference:NR_003287.4 Start:4599 End:4606
Model Median
Control
Saporin
Mean Intensity
log10 (Dwell Time)
4599 GTACG
4600 TACGA
4601 ACGAG
4602 CGAGA
4603 GAGAG
4604 AGAGG
4605 GAGGA
Reference position

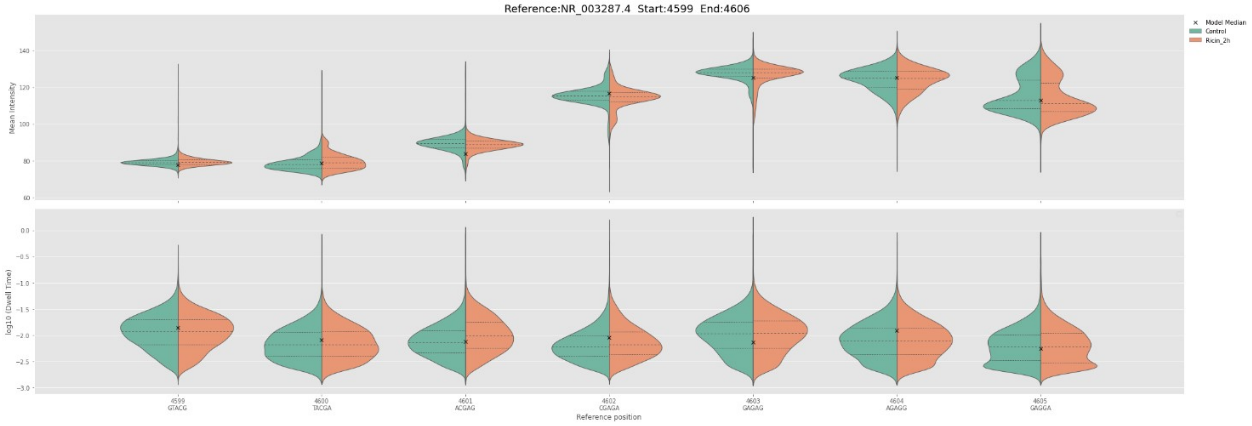
Reference:NR_003287.4 Start:4599 End:4606
Model Median
Control
Ricin_2h
Mean Intensity
log10 (Dwell Time)
4599 GTACG
4600 TACGA
4601 ACGAG
4602 CGAGA
4603 GAGAG
4604 AGAGG
4605 GAGGA
Reference position

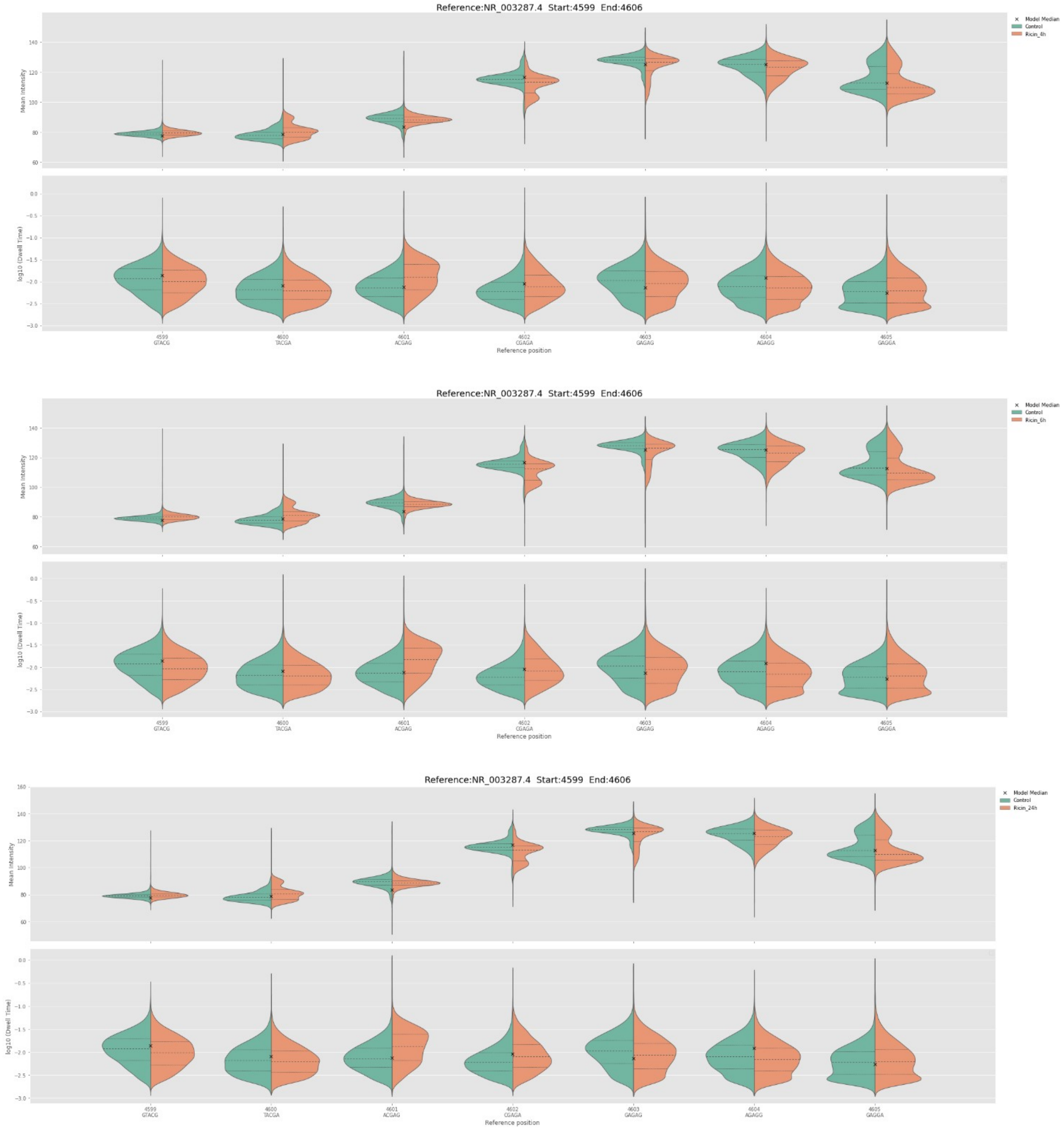

Figure S3. Fig. 5 Extended to All Conditions.

Figure S3. shows time dependence of the effects on charge density and dwell time, with saporin and earlier timepoints showing more similarity to control reads, and later ricin exposed time points showing greater changes. .

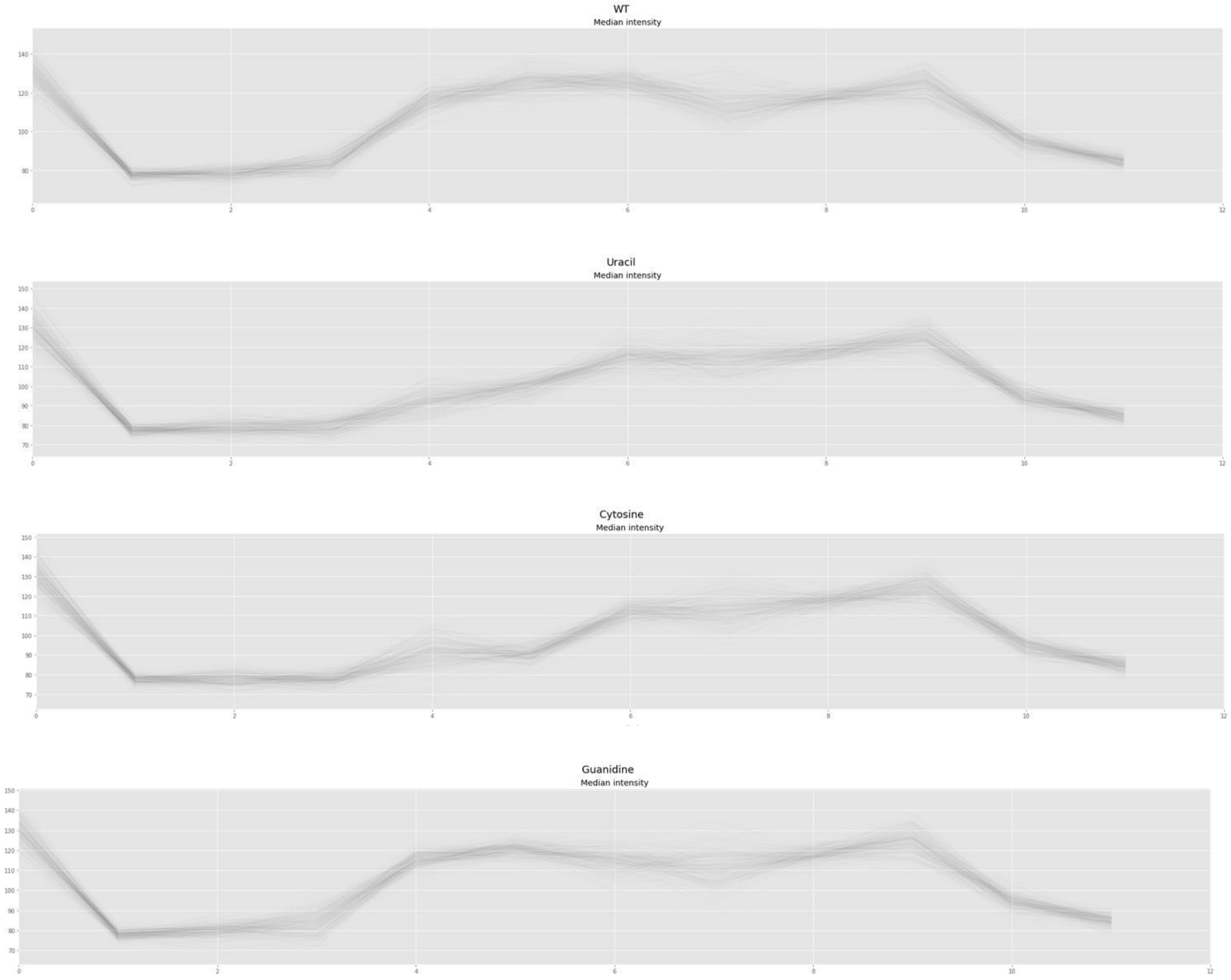

Figure S4. Fig. 6 extended with all four nucleotides.

Figure S4. shows Nanocompore simulated charge intensities for reads with all four bases at base 4605. Cytosine has a decrease in charge similar to uracil but plateaus between positions 4 and 5, whereas uracil continues decreasing. Guanidine appears similar to adenine, but at position 5 peaks at around 120 charge intensity, whereas adenine peaks nearer 130.

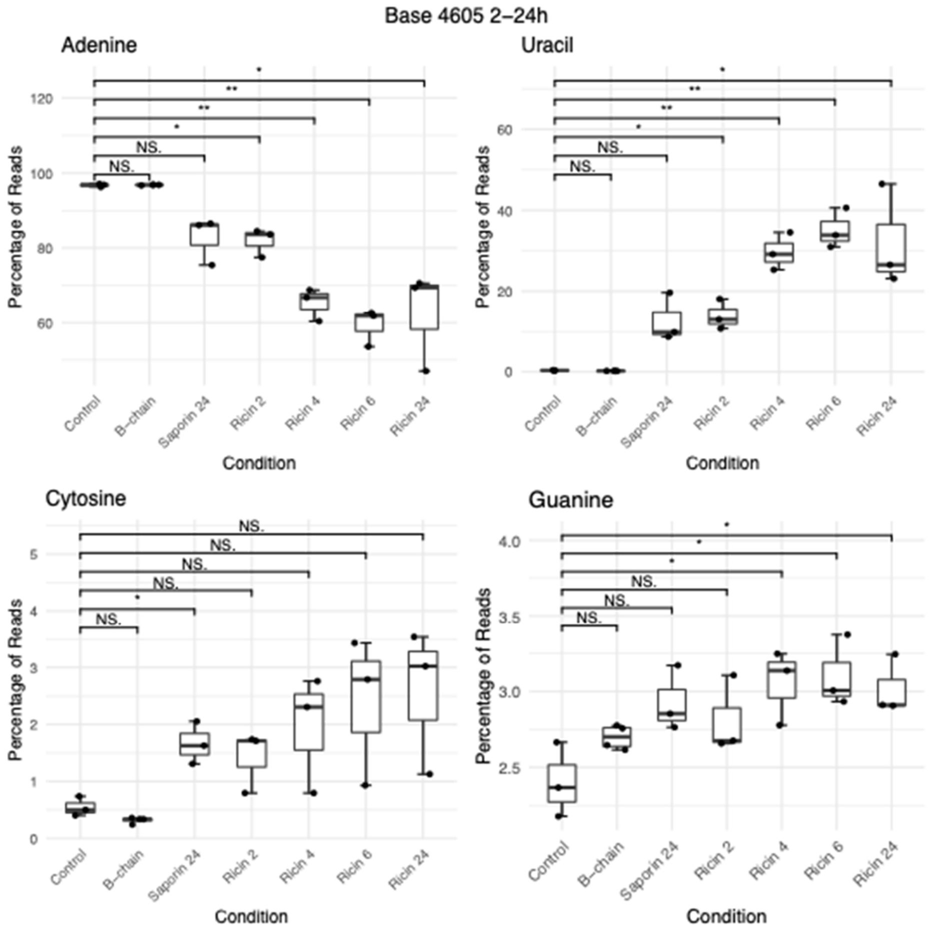

Figure S5. Boxplots showing a time course (2 – 24 h) of the percentage of nucleotide composition at position A4605 in 28S rRNA from A549 cells following exposure to vehicle control, ricin B-chain, saporin or pure ricin. either control, B-chain (6 h), or saporin at 24 h or ricin at 2, 4 ,6 and 24 h post-exposure. Pairwise t-tests assessing control to each exposure were used to perform significance testing.

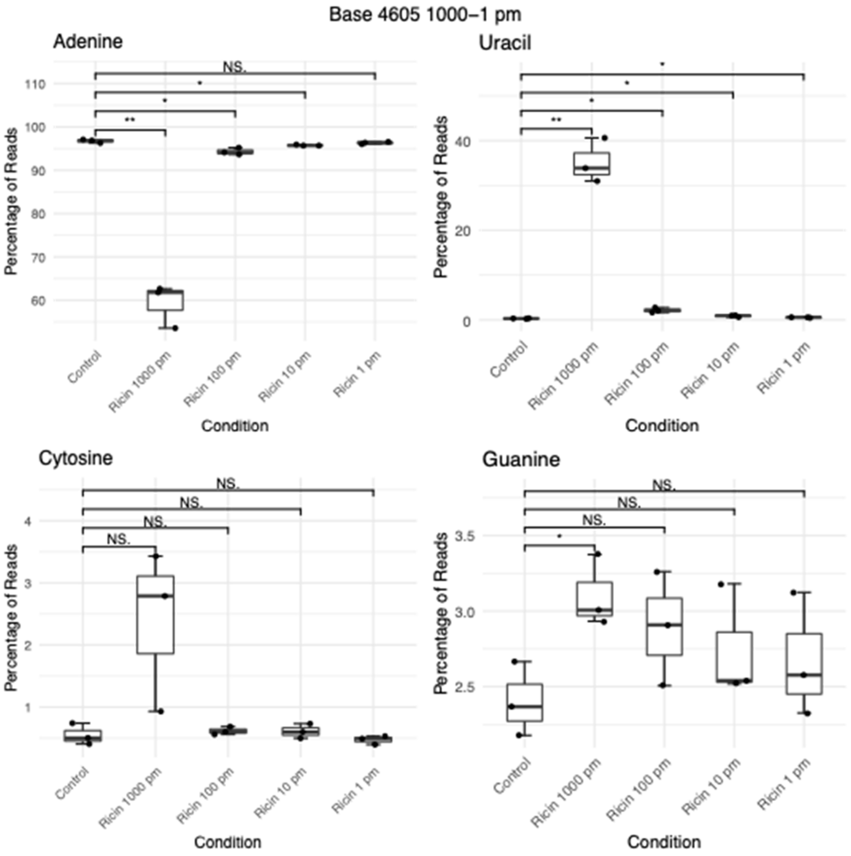

Figure S6. Boxplots showing percentage of nucleotide composition at position A4605 in 28S rRNA from A549 cells exposed to differing concentrations of ricin (1 – 1000 pm; 6 h). Pairwise t-tests assessing control to each exposure were used to perform significance testing.

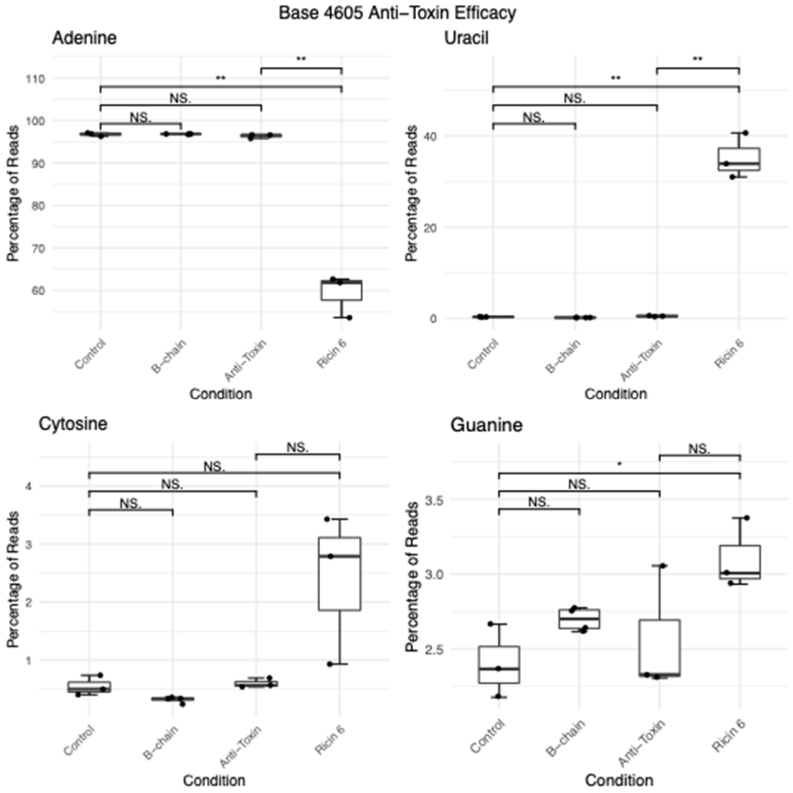

Figure S7. Boxplots showing the percentage of nucleotide composition at position A4605 in 28S rRNA from A549 cells following exposure to vehicle control, ricin B-chain (1nm, 6h), ricin (1nm, 6h), ricin and antitoxin (1nm ricin, neutralising dose antitoxin, 6h). Pairwise t-tests assessing control to each exposure were used to perform significance testing additionally with ricin and, antitoxin against ricin only.
